# Prognostic-Related Metabolic Score for Survival Prediction in Early-Stage Endometrioid Endometrial Cancer: A Multi-Center and Retrospective Study

**DOI:** 10.3389/fmed.2022.830673

**Published:** 2022-04-28

**Authors:** Zizhuo Wang, Kun Song, Jingbo Liu, Qing Zhang, Chuyao Zhang, Beibei Wang, Yu Fu, Yu Wang, Shuzhong Yao, Congjian Xu, Min Xia, Ge Lou, Jihong Liu, Bei Lin, Jianliu Wang, Weidong Zhao, Jieqing Zhang, Wenjun Cheng, Hongyan Guo, Ruixia Guo, Fengxia Xue, Xipeng Wang, Lili Han, Xia Zhao, Xiaomao Li, Ping Zhang, Jianguo Zhao, Jiezhi Ma, Qin Yao, Wenting Li, Xiaohang Yang, Yong Fang, Gang Chen, Kezhen Li, Yuanming Shen, Chaoyang Sun, Beihua Kong

**Affiliations:** ^1^Key Laboratory of the Ministry of Education, Cancer Biology Research Center, Tongji Hospital, Tongji Medical College, Huazhong University of Science and Technology, Wuhan, China; ^2^Department of Gynecology and Obstetrics, Tongji Hospital, Tongji Medical College, Huazhong University of Science and Technology, Wuhan, China; ^3^Department of Obstetrics and Gynecology, Qilu Hospital, Cheeloo College of Medicine, Shandong University, Jinan, China; ^4^Department of Gynecologic Oncology, Sun Yat-sen University Cancer Center, Guangzhou, China; ^5^Women’s Hospital, School of Medicine, Zhejiang University, Hangzhou, China; ^6^Department of Obstetrics and Gynecology, The First Affiliated Hospital, Sun Yat-sen University, Guangzhou, China; ^7^Department of Gynecology, Obstetrics and Gynecology Hospital of Fudan University, Shanghai, China; ^8^Department of Obstetrics and Gynecology, The Affiliated Yantai Yuhuangding Hospital of Qingdao University, Yantai, China; ^9^Department of Gynecology Oncology, Harbin Medical University Cancer Hospital, Harbin, China; ^10^Department of Obstetrics and Gynecology, ShengJing Hospital of China Medical University, Shenyang, China; ^11^Peking University People’s Hospital, Beijing, China; ^12^Division of Life Sciences and Medicine, The First Affiliated Hospital of USTC, University of Science and Technology of China, Hefei, China; ^13^Department of Gynecologic Oncology, Guangxi Medical University Cancer Hospital, Nanning, China; ^14^The First Affiliated Hospital of Nanjing Medical University, Nanjing, China; ^15^Peking University Third Hospital, Beijing, China; ^16^Department of Obstetrics and Gynecology, The First Affiliated Hospital of Zhengzhou University, Zhengzhou, China; ^17^Department of Obstetrics and Gynecology, Tianjin Medical University General Hospital, Tianjin, China; ^18^Department of Obstetrics and Gynecology, Xinhua Hospital, Shanghai Jiao Tong University School of Medicine, Shanghai, China; ^19^Department of Gynecology, People’s Hospital of Xinjiang Uygur Autonomous Region, Ürümqi, China; ^20^Development and Related Disease of Women and Children Key Laboratory of Sichuan Province, Key Laboratory of Birth Defects and Related Diseases of Women and Children, Ministry of Education, Department of Gynecology and Obstetrics, West China Second Hospital, Sichuan University, Chengdu, China; ^21^Department of Obstetrics and Gynecology, The Third Affiliated Hospital, Sun Yat-sen University, Guangzhou, China; ^22^Department of Gynecology, The Second Hospital of Shandong University, Jinan, China; ^23^Department of Gynecologic Oncology, Tianjin Central Hospital of Gynecology and Obstetrics, Affiliated Hospital of Nankai University, Tianjin, China; ^24^Tianjin Clinical Research Center for Gynecology and Obstetrics, Tianjin, China; ^25^Branch of National Clinical Research Center for Gynecology and Obstetrics, Tianjin, China; ^26^Department of Obstetrics and Gynecology, The Third Xiangya Hospital, Central South University, Changsha, China; ^27^Department of Obstetrics and Gynecology, The Affiliated Hospital of Qingdao University, Qingdao, China

**Keywords:** metabolic status, partial metabolic disorders, early-stage endometrioid endometrial cancer, progression-free survival time, ECPRM Score

## Abstract

**Objective:**

Patients with endometrial cancer (EC) combined with metabolic syndrome (MetS) have a worse prognosis than those without MetS. This study aimed to investigate whether partial metabolic disorder significantly influenced early-stage endometrioid EC (EEC) survival and searched for a more efficient method to evaluate metabolic status.

**Methods:**

This is a nationwide, multicenter cohort study that included 998 patients with primary early-stage EEC from 2001 to 2018. Patients were divided into different metabolic groups based on the diagnostic criteria of the Chinese Medical Association (CDC). The progression-free survival (PFS) time was compared between various metabolic status. Meanwhile, we established an EC Prognostic-Related Metabolic Score (ECPRM Score) to explore the association of the severity of metabolic status and early-stage EEC PFS. A nomogram was established for predicting PFS, which was externally validated in a testing set that includes 296 patients.

**Results:**

A partial metabolic disorder, as well as MetS, was an independent risk factor of poor survival of patients with early-stage EEC [hazard ratio (HR) = 7.6, 95% CI = 1.01–57.5, *p* < 0.05]. A high ECPRM Score was associated with lower PFS (HR = 2.1, 95% CI = 1.05–4.0, *p* < 0.001). The nomogram, in which the ECPRM Score contributed most to the prognosis, exhibited excellent discrimination of survival supported by the internal and external validations. In addition, the calibration curve supports its robust predicting ability.

**Conclusion:**

Even though they do not meet the criteria of MetS, partial metabolic disorders were also associated with adverse outcomes in early-stage EEC. The ECPRM Score is beneficial for clinicians to evaluate the severity of metabolic abnormalities and guide patients to ameliorate the poor prognosis of metabolic disorders.

## Introduction

Endometrial cancer (EC) is the sixth most common malignancy in women worldwide (1) and the most common gynecologic malignancy in the United States (2). In China, the incidence of EC is also increasing rapidly, and the age of onset is becoming younger (3). A metabolic disorder, one of the most important comorbidities of EC, significantly shortens the survival time of patients with EC (4).

Metabolic syndrome (MetS) is a complex disorder that is considered a worldwide epidemic in western countries and China (5). There is a strong association between EC and MetS. Mounting studies revealed that women with MetS are estimated to have a higher EC risk when compared to those without MetS (6–11). MetS not only increases the prevalence of EC but also results in poor prognosis. Kokts-Porietis et al. identified that MetS, in particular central adiposity, was associated with worse overall and disease-free survival in EC among 540 survivors in Canada (12). Nevertheless, few studies with a large sample size in the Asia-Pacific population have confirmed the relationship between MetS and EC prognosis.

Although MetS contains many components and clinical implications, there are still no universally accepted or clearly defined diagnostic criteria (5). In 2001, the National Cholesterol Education Program published the Adult Treatment Panel III that includes a set of criteria that included waist circumference, blood lipids, blood pressure, and fasting blood glucose (BG) (13), which is the basis of the 2005 International Diabetes Federation (IDF) criteria that introduced abdominal obesity as a prerequisite of the diagnosis of MetS (14). A recent Italian study showed that obesity negatively influenced the preoperative and postoperative tumor grading concordance, leading to a lower pathological diagnostic accuracy, and is adverse for EC survival (15). However, the epidemiological characteristics of MetS vary with region, lifestyle pattern, and socioeconomic status. According to the Chinese Medical Association (CDC) criterion of MetS, body mass index (BMI), hyperglycemia, hypertension, and dyslipidemia are the four main components of MetS in the Asian population. The dichotomous nature of these criteria makes it difficult to measure the severity of metabolic abnormalities since a high-risk state exists among the four main components (16, 17).

Moreover, MetS has been criticized for not providing additional risk information beyond its individual components (18–20). Meanwhile, partial metabolic abnormalities, which have not yet reached the diagnostic criteria for MetS, existed in a significant number of patients with EC. However, few studies have concentrated on the impacts of partial metabolic disorders on the prognosis of these patients with EC. Therefore, we attempted to establish a continuous metabolic disorder score to evaluate the associations between metabolic disorder and prognosis in EC.

Endometrioid endometrial carcinomas (EECs) constitute approximately 85% of newly diagnosed cases (21). Simultaneously, the symptoms appear early in the course of EC, which explains why most patients have early-stage disease at presentation (22). Accordingly, patients with early-stage EEC were the majority in EC. In this study, we conducted a nationwide retrospective study that included 998 early-stage EEC patients, aiming at the Asia-Pacific population, to explore the relationship between MetS or partial metabolic disorders and survival. We demonstrated that partial metabolic disorders and MetS were both associated with adverse clinical outcomes. Moreover, we put forward a novel method to calculate EC Prognosis-Related Metabolic Score (ECPRM Score) and validated its remarkable ability to predict early-stage EEC survival in an external testing set.

## Materials and Methods

### Study Design and Data Collection

We retrospectively collected clinicopathological data from patients with early-stage EEC in 13 hospitals around China from 2001 to 2018 for the training set (trainset, *n* = 998). We involved patients were with complete medical records and follow-up information. Patients who received radiotherapy or chemotherapy before surgery or with other malignant tumors were excluded. For the testing set (testset), 296 patients with early-stage EEC from another hospital were enrolled. They also possessed complete clinicopathological data and follow-up information. This study was approved by the Institutional Review Boards of all included hospitals.

Sociodemographic data that included age (<50, ≥50) and family history (no, yes) were gathered from the electronic medical record. Disease-related data, such as pathologic grade [low grade (G1 and 2), high grade (G3 and 4)] and lymph-vascular space invasion (LVSI; no, yes), were collected in postoperative pathological reports. The endpoint index was progression-free survival (PFS) time, which was defined as the time from the date of the primary surgery to the date of the last follow-up or recurrence.

### Diagnostic Criteria of Metabolic Syndrome

The MetS was defined according to the recommendations of the Diabetes Society of the CDC (23): (1) overweight and/or obese: BMI ≥25 kg/m^2^, (2) hyperglycemia: fasting postprandial BG ≥6.1 mmol/L, (3) hypertension: systolic/diastolic blood pressure (SBP/DBP) ≥140/90 mmHg, (4) dyslipidemia: triglycerides ≥1.7 mmol/L, and/or high-density lipoprotein cholesterol (HDL) <1.0 mmol/L (women). The diagnosis of MetS was based on the existence of at least three abnormal findings out of the four mentioned above.

Each participant included in the study was grouped into one of the three categories: (1) patients who were free of metabolic disorder [free metabolic disorder in patients (FMDPs)], (2) patients with one or two metabolic disorders [partial metabolic disorder in patients (PMDPs)], (3) with three or four metabolic abnormal findings [metabolic syndrome in patients (MetSPs)].

### Endometrial Cancer Prognostic-Related Metabolic Score

We calculated the ECPRM Score using a method of confirmatory factor analyses (24, 25). We included five identified MetS components: BMI, SBP, HDL, triglycerides, and BG in the ECPRM Score. According to the CDC guidelines above, BMI, which is a more suitable indicator of MetS in the Asia-Pacific population, was used to define overweight and obesity. For the trainset (*n* = 998), triglycerides were log-transformed, the inverse of HDL was used, and all variables were standardized (mean = 0, SD = 1) over the entire sample. Then, a one-factor model formed based on all confirmatory factor analyses was performed by the “lavaan” R package. The factor loadings from the five MetS components were determined and used to generate equations for computing a MetS severity score. The resulting score has a standard normal distribution that operates as a “z score,” which we named ECPRM Score (Supplementary Table 1). Since all variables contributing to the scores were standardized (mean = 0), we categorized the patients with ECPRM Score > 0 as the high score group and those with ECPRM Score ≤ 0 as the low score group.

### Statistical Analysis

All statistical analyses were performed using R version 3.6.3^1^ and its appropriate packages. A two-sided *p* < 0.05 was considered statistically significant. For baseline data analysis in the trainset, we applied the chi-square test to compare categorical variables in FMDP, PMDP, and MetSP groups. The ANOVA analysis was applied to compare the scores of the three groups. Kaplan–Meier survival analysis was conducted to explore the difference in PFS in three groups, and the *p* was used to identify significant differences between groups. The multivariate Cox proportional hazard models and the Schoenfeld residuals test were conducted to, respectively, evaluate the hazard ratios (HRs) and proportional hazard assumptions of the clinicopathological variables and metabolic groups or score groups. The PFS possibilities of 5 and 7 years were quantified according to the nomogram based on the multivariate Cox regression analysis that includes score groups. The concordance index (C-index) and a calibration curve were used to measure discriminative capacity. For testset, ECPRM Score was calculated according to the formula derived from the trainset. The Kaplan–Meier survival analysis was conducted to explore the difference between the high-score group and low-score group. Multivariate Cox regression analyses were also employed as above. We conducted a predictive model that includes risk score group using a nomogram in the trainset and validated in the testset.

## Results

### Demographics and Clinical Characteristics

In total, 998 patients with primary early-stage EEC who underwent hysterectomy were retrospectively analyzed in the trainset. The patients were partitioned into FMDP, PMDP, and MetSP groups according to the CDC (see section “Materials and Methods”). In total, 339 patients (34.0%) were diagnosed as MetS (MetSP). Notably, 530 (53.1%) patients were present as partial metabolic abnormality (PMDP), while only 129 patients (12.9%) had no metabolic disorder (FMDP). This shows that most patients with early-stage EEC are in the state of PMDP, so it is essential to survey the prognostic features of these populations.

The characteristics of the included patients are listed in Table 1. A total of 826 (82.8%) patients were diagnosed with low grade (G1 and G2), while 172 (17.2%) were with high grade (G3 and G4). The patients over 50 years old accounted for 74.3%. The majority of patients were without a family history (84.4%). Only a small proportion of patients had LVSI (6.8%).

**TABLE 1:** Characteristics of free metabolic disorder in patients (FMDP), partial metabolic disorder in patients (PMDP), metabolic syndrome in patients (MetSP) patients in the training set (*n* = 998).

Characteristics	Patient no. (%)				
	Total (*n* = 998)	FMDP (*n* = 129)	PMDP (*n* = 530)	MetSP (*n* = 339)	*P*-value
**Grade**					0.780
G1 and 2	826 (82.8)	104 (80.6)	440 (83.0)	282 (83.2)	
G3 and 4	172 (17.2)	25 (19.4)	90 (17.0)	57 (16.8)	
**Age group**					0.087
Age < 50	256 (25.7)	43 (33.3)	134 (25.3)	79 (23.3)	
Age > = 50	742 (74.3)	86 (66.7)	396 (74.7)	260 (76.7)	
**Family history**					0.089
No	842 (84.4)	117 (90.7)	440 (83.0)	285 (84.1)	
Yes	156 (15.6)	12 (9.3)	90 (17.0)	54 (15.9)	
**Lymph-vascular space invasion (LVSI)**					0.333
No	930 (93.2)	118 (91.5)	491 (92.6)	321 (94.7)	
Yes	68 (6.8)	11 (8.5)	39 (7.4)	18 (5.3)	

*Values of p are based on the chi-square test among three groups in the clinical characteristics. MetSP, patients with metabolic syndrome; PMDP, patients with partial metabolic abnormality; FMDP, patients with free metabolic disorder.*

### Partial Metabolic Disorder in Patient Was an Independent Risk Factor in Predicting Early-Stage Endometrioid Endometrial Carcinoma Prognosis

**FIGURE 1:**
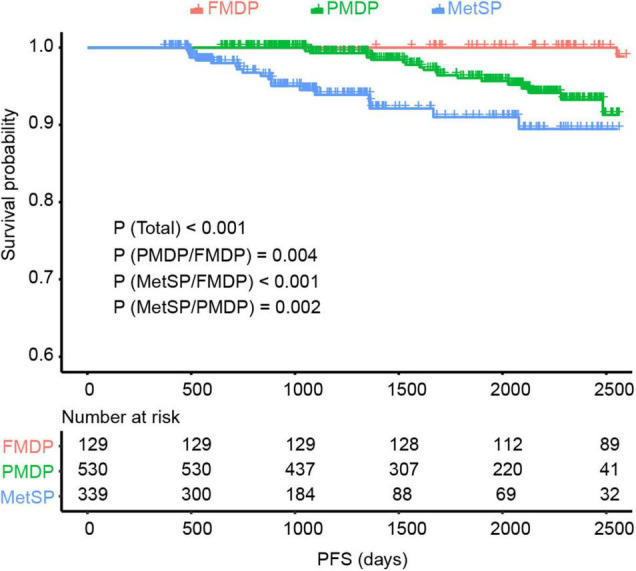
The Kaplan–Meier survival analysis showed significant differences between the three groups [*p* < 0.001 (Total)] and pairwise comparisons {*p* = 0.004 [partial metabolic disorder patients (PMDP)/free metabolic disorder in patients (FMDPs)], *p* < 0.001 [metabolic syndrome in patients (MetSP)/FMDP], *p* = 0.002 [metabolic syndrome in patients (MetSPs)/PMDP]} also showed noteworthy differences in EC. The MetSP group had the best progression-free survival (PFS), followed by PMDP group and then the FMDP group.

**FIGURE 2:**
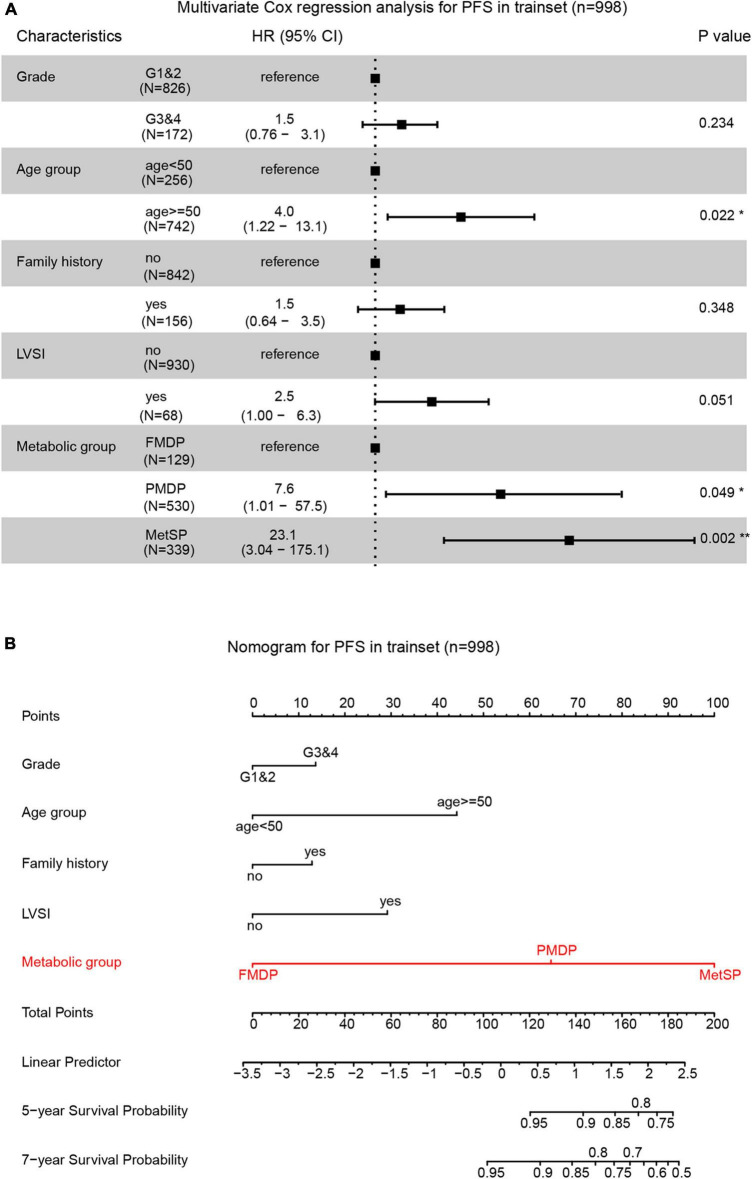
Partial metabolic disorder patient (PMDP) was an independent risk factor in predicting early-stage EEC prognosis. **(A)** A forest plot of the multivariate Cox regression analysis showed PMDPs and metabolic syndrome in patients (MetSPs) were significantly associated with shorter PFS. **(B)** A nomogram was built by integrating pathologic grade, age group, family history, lymph-vascular space invasion (LVSI), and metabolic group and indicated the robust predicting ability of the metabolic group.

We explored the impact of metabolic disorders on PFS of early-stage EEC patients using the Kaplan–Meier survival analysis. Significant differences were found between the three groups, and pairwise comparisons also showed noteworthy differences. The FMDP group had the best prognosis, followed by the PMDP group and then the MetSP group (*p* = 0.004 (PMDP/FMDP), *p* < 0.001 (MetSP/FMDP), *p* = 0.002 (MetSP/PMDP), Figure 1). Therefore, patients with PMDP and MetS were significantly associated with worse PFS than FMDP. It is noteworthy that PMDP accounted for the majority of early-stage EEC. To further investigate whether PMDP was an independent factor for adverse survival, we conducted multivariate Cox regression analysis among pathologic grade, age group, family history, LVSI, and metabolic group. To our surprise, after adjusting the other clinicopathological variables, PMDP was still an independent risk factors for poor prognosis (HR = 7.6, 95% CI = 1.01–57.5, *p* < 0.05, Figure 2A). Further, a nomogram was built based on the multivariate Cox regression analysis above, indicating the robust predicting ability of metabolic disorder with the greatest contribution (Figure 2B).

### Endometrial Cancer Prognostic-Related Metabolic Score Possessed the Remarkable Ability in Predicting Progression-Free Survival in Early-Stage Endometrioid Endometrial Carcinoma

In the above section, we have recognized that patients in the PMDP group, who did not meet the diagnostic criteria of MetS, were also associated with worse survival than the FMDP group, which indicates that the diagnostic criteria of MetS could not completely reflect the influence of metabolic disorder on the prognosis of early-stage EEC. In light of this, we further tried to establish a continuous ECPRM Score to precisely evaluate the severity of the metabolic disorder in patients with EC by confirmatory factor analysis. We calculated the ECPRM Score according to the formula based on the five identified MetS components: BMI, SBP, HDL, triglycerides, and BG (see section “Materials and Methods”). The ECPRM Score was highest in the MetSP group, followed by the PMDP and the FMDP groups, elucidating that the ECPRM Score was susceptible to metabolic status (Supplementary Figure 1).

**FIGURE 3:**
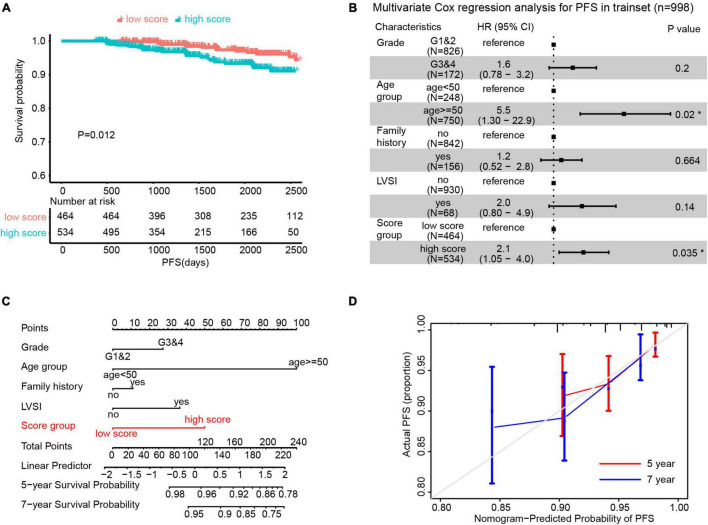
Endometrial Cancer Prognostic-Related Metabolic (ECPRM) Score possessed the remarkable ability in predicting PFS in early-stage EEC. **(A)** The Kaplan-Meier survival analysis revealed the strong association between high ECPRM Score and shorter PFS in the trainset. **(B)** A forest plot of the multivariate Cox regression analysis showed that the high ECPRM Score was significantly associated with shorter PFS in the trainset. **(C)** A nomogram was built by integrating pathologic grade, age group, family history, LVSI, and score group, indicating the robust predicting ability of a high ECPRM Score. **(D)** A calibration plot for the nomogram was conducted based on the trainset, and the favorable agreements were shown between the actual and estimated probability of PFS in 5 and 7 years.

Since all variables contributing to the scores were standardized (mean = 0), we defined the patients with ECPRM Score > 0 as the high score group and those with ECPRM Score ≤ 0 as the low score group. The Kaplan–Meier curve showed that patients in the high score group survived significantly longer than the low score group (Figure 3A). Through the multivariable Cox regression analysis after the correction for other clinicopathological variables, the high ECPRM Score remains an independent risk factor for poor prognosis (HR = 2.1, 95% CI = 1.05–4.0, *p* < 0.05, Figure 3B). To establish a more user-friendly model for predicting the survival outcomes, we combined pathologic grade, age group, family history, LVSI, and ECPRM Score group to conduct a nomogram based on the multivariable Cox regression analysis. Similarly, the nomogram revealed that a high ECPRM Score significantly contributed to the early-stage EEC prognosis (Figure 3C). Then, to evaluate our nomogram’s ability to predict PFS in patients with early-stage EEC, we conducted a calibration plot for our model. A C-index of 0.73 was achieved, and the favorable agreements were shown between the actual and the estimated probability of PFS in 5 and 7 years (Figure 3D).

### The Predictive Value of the Endometrial Cancer Prognostic-Related Metabolic Score Was Validated in an External Cohort

**FIGURE 4:**
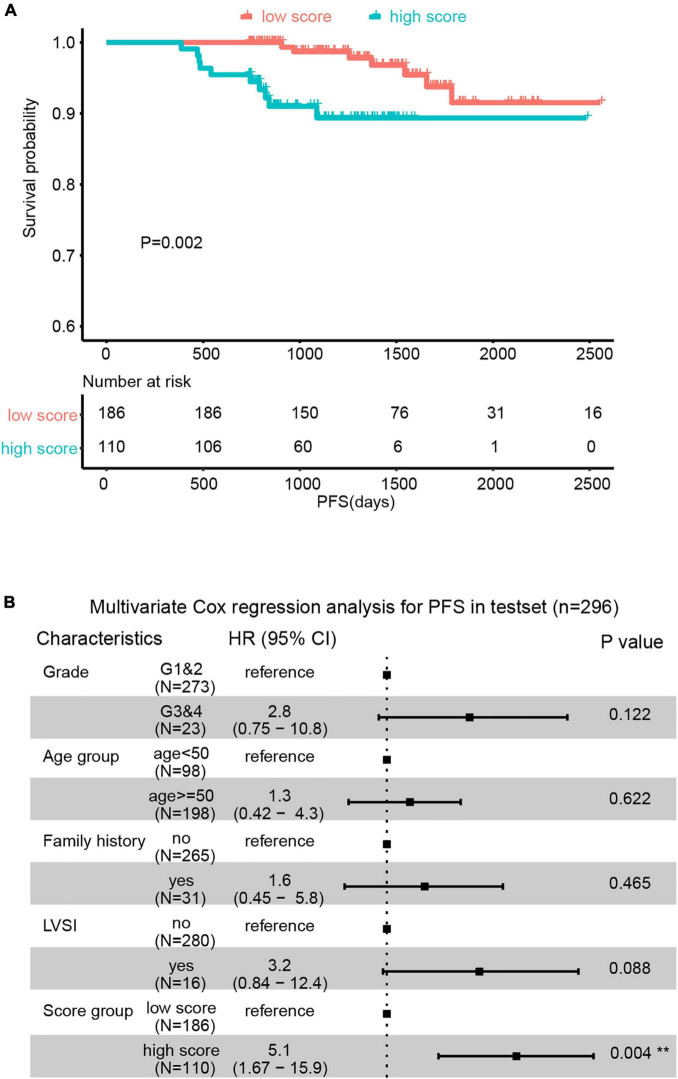
The predictive value of Endometrial Cancer Prognostic-Related Metabolic (ECPRM) Score was validated in an external cohort. **(A)** Kaplan-Meier survival analysis revealed the strong association between high ECPRM Score and shorter PFS in the testset. **(B)** Forest plot of multivariate Cox regression analysis showed high ECPRM Score was significantly associated with shorter PFS in the testset.

The external validation was performed in the testset (*n* = 296). All patients obtained the ECPRM Score in terms of the computing formula (Supplementary Table 1). Patients with ECPRM Score > 0 were classified as high score groups and those with ECPRM Score ≤ 0 were low score groups. The PFS possibility of the high score group was distinguishingly shorter than that of the low score group (Figure 4A). The multivariable Cox regression analysis exhibited a remarkable association between the high score group and poor prognosis (Figure 4B). When applied to the original model, the testset produced a C-index of 0.7, confirming the satisfactory accuracy in predicting the PFS possibility of the nomogram of external validation.

## Discussion

In the current study, we identified that partial metabolic disorder and MetS both significantly influenced early-stage EEC prognosis. As expected, a worse prognosis was observed in the MetSP group when compared with the FMDP and PMDP groups. Unexpectedly, the PFS of the PMDP group was longer than the MetSP group and shorter than the FMDP group, and the severer of metabolic abnormalities, the shorter the PFS. PMDP and MetSP were both independent risk factors for poor survival in early-stage EEC. It is noteworthy that the PMDP, which accounted for the majority of our dataset, was not accurate since it was a qualitative concept rather than a quantitative one (20). Nevertheless, controversy existed over various sets of MetS and partial metabolic disorder criteria (26–29). The different degrees of partial metabolic disorder determined different prognoses (30).

Therefore, to evaluate the quantitative connection between metabolic disorder and survival, we demonstrate a novel scoring approach of metabolic abnormalities status that could effectively predict early-stage EEC prognosis. The ECPRM Score was based on accurate indexes from laboratory tests without considering subjective items, such as self-descriptive previous history. Every patient owned an exact ECPRM Score representing their metabolic status. Compared to simple classification based on FMDP/PMDP/MetSP, the ECPRM Score, which characterized metabolic status more correctly and standardized, was beneficial to investigate the association between the metabolic status and prognosis of patients with early-stage EEC. In our dataset, the Kaplan–Meier curve showed that the ECPRM Score was significantly associated with worse PFS. After eliminating the effect of pathologic grade, age group, family history, and LVSI, the ECPRM Score group was still identified as a significant prognostic factor.

To our knowledge, this investigation is the first study using the CDC’s recommended diagnostic criteria of MetS to explore its association with survival outcomes in early-stage EEC. Our findings are generally consistent with prior literature using other criteria that observed the association of worse prognosis with MetS among patients with EC (12, 31, 32). Kokts-Porietis et al. used the country- and sex-specific waist circumference criteria of MetS (33) (presence of ≥three of the following components: waist circumference ≥88 cm, fasting BG levels ≥100 mg/dl, blood triglycerides ≥150 mg/dl, HDL cholesterol <50 mg/dl, or self-reported hypertension and/or hypertension medication use) to assess the association with overall and disease-free survival of EC in North Americans (12). The authors noted that patients with EC with high waist circumference had 2.12 (95% CI = 1.8–3.8) times the hazard of overall survival time when compared to those with low waist circumference (12). However, waist circumference is not a routinely useful indicator of MetS in the Asia-Pacific population. As we know, the appearance of metabolic disorders varied from races (24). Asia-Pacific adults were more susceptible to overall obesity and fat distribution on metabolic risk factors than the other racial/ethnic population (34). Furthermore, this was the reason that we brought BMI instead of waist circumference into our study. A study based on the 2010 China Non-communicable Disease Surveillance providing the latest estimate of the national prevalence of MetS in Asia used the lower cutoff points for abdominal obesity according to the International Obesity Task Force criteria for the Asian-Pacific population (35). The BMI in the Asia-Pacific population is significantly lower than those in the white population. Thus, we utilized the diagnostic criteria of CDC, putting BMI at a vital position instead of waist circumference to define MetS in a Chinese context. In light of this, the conclusion has strong generalizability in the Asia-Pacific population. We confirmed that the MetS status shortens the survival time and partial metabolic disorder is also associated with adverse clinical outcomes of patients with early-stage EEC.

Since the impact of partial metabolic disorder on PFS, we considered it necessary to develop a new quantitative method of evaluating the metabolic status, which is more effective in forecasting early-stage EEC prognosis. The principle of the ECPRM Score is confirmatory factor analysis, as described previously (24, 25). In the trainset, the high score was an independent risk factor for poor survival. We conducted a nomogram containing pathologic grade, age group, family history, LVSI, and score group and demonstrated that high score contributes most to the adverse prognosis. The model was internally validated by the C-index (0.73) and the calibration curves were 5 and 7 years. Meanwhile, the testset also revealed a strong association between the high score and the shorter PFS. The C-index (0.7) confirmed the efficiency of the nomogram. To our knowledge, this investigation is the first study to use a method of continuous score to describe the severity of metabolic status and explore its association with early-stage EEC survival outcomes.

This study has several key strengths. First, we identified that the partial metabolic disorder also influenced significantly on PFS and MetS. Subsequently, we exploited a novel score system to quantitate metabolic status accurately. This is the first study to employ the ECPRM Score to evaluate the impact of metabolic status on the early-stage EEC prognosis. Second, the epidemiological characteristics of MetS vary greatly with region, lifestyle pattern, and socioeconomic status, and the manifestation of metabolic abnormality is quite different between Asia-Pacific and the western population. The relationship between metabolic abnormalities and EC prognosis has rarely been reported in the Asia-Pacific population. This investigation included patients from 14 medical centers around China. Therefore, it is more than necessary to adopt the diagnostic criteria of MetS that is suitable for the Asia-Pacific population. Finally, it is vital that early intervention should be applied among PMDP with high risk since the influence of partial metabolic disorder on early-stage EEC survival was significant, same as MetS.

So that, through our ECPRM Score, we identified the high-risk patients, who did not meet the diagnostic criteria for MetS, but the metabolic disorders of whom also had an impact on the prognosis of the early-stage EEC. There is a limitation to the current study. Timely surgical treatment is the most important factor influencing the early-stage EEC prognosis (36–39). However, all patients received radical operation in the study. Thus, we could not investigate the impact of surgery on the early-stage EEC survival. The importance of surgery should be emphasized.

## Conclusion

Our study confirmed that MetS, as a vital complication, was significantly related to shorter PFS of patients with early-stage EEC. Meanwhile, we put forward the concept that partial metabolic disorder was also significantly associated with early-stage EEC prognosis. We utilized the ECPRM Score to evaluate the metabolic status and explore its prognostic value of early-stage EEC survival for the first time. This study is helpful for clinicians to reasonably evaluate the metabolic status of patients with early-stage EEC and prolong the survival by intervening the metabolic status.

## Data Availability Statement

The original contributions presented in the study are included in the article/Supplementary Material, further inquiries can be directed to the corresponding author/s.

## Ethics Statement

The studies involving human participants were reviewed and approved by the Institutional Review Board of Huazhong University of Science and Technology. The patients/participants provided their written informed consent to participate in this study.

## Author Contributions

BK and CS worked on the design of the study and supervised the whole study. ZW, KS, JinL, QZ, CZ, BW, YuF, YW, SY, CX, MX, GL, JihL, BL, JW, WZ, JieZ, WC, HG, RG, FX, XW, LH, XZ, XL, PZ, JiaZ, JM, QY, WL, XY, YoF, GC, KL, YS, CS, and BK conducted the data collection. ZW, KS, and JinL performed the data analyses and manuscript writing. All authors contributed to the article and approved the submitted version.

## Conflict of Interest

The authors declare that the research was conducted in the absence of any commercial or financial relationships that could be construed as a potential conflict of interest.

## Publisher’s Note

All claims expressed in this article are solely those of the authors and do not necessarily represent those of their affiliated organizations, or those of the publisher, the editors and the reviewers. Any product that may be evaluated in this article, or claim that may be made by its manufacturer, is not guaranteed or endorsed by the publisher.

## Footnotes

1
http://www.R-project.org

## Supplementary Material

Supplementary Figure 1 ANOVA analysis revealed significant differences in endometrial cancer prognostic-related metabolic (ECPRM) Score of the free metabolic disorder in patient (FMDP), partial metabolic disorder patient (PMDP), and metabolic syndrome in patient (MetSP) group in the trainset.

## References

[1] Bray F Ferlay J Soerjomataram I Siegel RL Torre LA Jemal A. Global cancer statistics 2018: GLOBOCAN estimates of incidence and mortality worldwide for 36 cancers in 185 countries. *CA Cancer J Clin.* (2018) 68:394–424. 10.3322/caac.21492 30207593

[2] Siegel RL Miller KD Jemal A. Cancer statistics, 2020. *CA Cancer J Clin.* (2020) 70:7–30. 10.3322/caac.21590 31912902

[3] He S Fang X Xia X Hou T Zhang T. Targeting CDK9: a novel biomarker in the treatment of endometrial cancer. *Oncol Rep.* (2020) 44:1929–38. 10.3892/or.2020.7746 32901849 PMC7551504

[4] Jiang Y Chen J Ling J Zhu X Jiang P Tang X Construction of a glycolysis-related long noncoding RNA signature for predicting survival in endometrial cancer. *J Cancer.* (2021) 12:1431–44. 10.7150/jca.50413 33531988 PMC7847640

[5] Kassi E Pervanidou P Kaltsas G Chrousos G. Metabolic syndrome: definitions and controversies. *BMC Med.* (2011) 9:48. 10.1186/1741-7015-9-48 21542944 PMC3115896

[6] Arthur RS Kabat GC Kim MY Wild RA Shadyab AH Wactawski-Wende J Metabolic syndrome and risk of endometrial cancer in postmenopausal women: a prospective study. *Cancer Causes Control.* (2019) 30:355–63. 10.1007/s10552-019-01139-5 30788634 PMC6886235

[7] Adambekov S Yi Y Fabio A Miljkovic I Edwards RP Lopa S Metabolic syndrome in endometrial cancer patients: systematic review. *Metab Syndr Relat Disord.* (2019) 17:241–9. 10.1089/met.2018.0106 30932741

[8] Passarello K Kurian S Villanueva V. Endometrial cancer: an overview of pathophysiology, management, and care. *Semin Oncol Nurs.* (2019) 35:157–65. 10.1016/j.soncn.2019.02.002 30867105

[9] Friedenreich CM Biel RK Lau DC Csizmadi I Courneya KS Magliocco AM Case-control study of the metabolic syndrome and metabolic risk factors for endometrial cancer. *Cancer Epidemiol Biomarkers Prev.* (2011) 20:2384–95. 10.1158/1055-9965.Epi-11-0715 21921255

[10] Esposito K Chiodini P Colao A Lenzi A Giugliano D. Metabolic syndrome and risk of cancer: a systematic review and meta-analysis. *Diabetes Care.* (2012) 35:2402–11. 10.2337/dc12-0336 23093685 PMC3476894

[11] Rosato V Zucchetto A Bosetti C Dal Maso L Montella M Pelucchi C Metabolic syndrome and endometrial cancer risk. *Ann Oncol.* (2011) 22:884–9. 10.1093/annonc/mdq464 20937645

[12] Kokts-Porietis RL McNeil J Nelson G Courneya KS Cook LS Friedenreich CM. Prospective cohort study of metabolic syndrome and endometrial cancer survival. *Gynecol Oncol.* (2020) 158:727–33. 10.1016/j.ygyno.2020.06.488 32600790

[13] Expert Panel on Detection, Evaluation, and Treatment of High Blood Cholesterol in Adults. Executive summary of the third report of the national cholesterol education program (NCEP) expert panel on detection, evaluation, and treatment of high blood cholesterol in adults (adult treatment panel III). *JAMA.* (2001) 285:2486–97. 10.1001/jama.285.19.2486 11368702

[14] Alberti KG Zimmet P Shaw J. The metabolic syndrome–a new worldwide definition. *Lancet.* (2005) 366:1059–62. 10.1016/s0140-6736(05)67402-8 16182882

[15] Capozzi VA Monfardini L Sozzi G Butera D Armano G Riccò M Obesity, an independent predictor of pre and postoperative tumor grading disagreement in endometrial cancer. *Eur J Obstet Gynecol Reprod Biol.* (2021) 262:160–5. 10.1016/j.ejogrb.2021.05.028 34022594

[16] Goodman E Daniels SR Meigs JB Dolan LM. Instability in the diagnosis of metabolic syndrome in adolescents. *Circulation.* (2007) 115:2316–22. 10.1161/CIRCULATIONAHA.106.669994 17420347 PMC2626638

[17] DeBoer MD Gurka MJ Woo JG Morrison JA. Severity of metabolic syndrome as a predictor of cardiovascular disease between childhood and adulthood: the Princeton Lipid research cohort study. *J Am Coll Cardiol.* (2015) 66:755–7. 10.1016/j.jacc.2015.05.061 26248997 PMC4612636

[18] Kahn R Buse J Ferrannini E Stern M. The metabolic syndrome: time for a critical appraisal: joint statement from the American diabetes association and the European association for the study of diabetes. *Diabetes Care.* (2005) 28:2289–304. 16123508 10.2337/diacare.28.9.2289

[19] Mozaffarian D Kamineni A Prineas RJ Siscovick DS. Metabolic syndrome and mortality in older adults: the cardiovascular health study. *Arch Intern Med.* (2008) 168:969–78. 10.1001/archinte.168.9.969 18474761

[20] Gurka MJ Golden SH Musani SK Sims M Vishnu A Guo Y Independent associations between a metabolic syndrome severity score and future diabetes by sex and race: the atherosclerosis risk in communities study and Jackson heart study. *Diabetologia.* (2017) 60:1261–70. 10.1007/s00125-017-4267-6 28378033 PMC5481783

[21] Bell DW Ellenson LH. Molecular genetics of endometrial carcinoma. *Annu Rev Pathol.* (2019) 14:339–67. 10.1146/annurev-pathol-020117-043609 30332563

[22] Amant F Moerman P Neven P Timmerman D Van Limbergen E Vergote I. Endometrial cancer. *Lancet.* (2005) 366:491–505. 10.1016/s0140-6736(05)67063-8 16084259

[23] Metabolic Syndrome Research Cooperative Group of Diabetes Society of Chinese Medical Association. Recommendations of diabetes society of Chinese medical association on metabolic syndrome. *Chin J Diabetes.* (2004) 5–10.

[24] Gurka MJ Lilly CL Oliver MN DeBoer MD. An examination of sex and racial/ethnic differences in the metabolic syndrome among adults: a confirmatory factor analysis and a resulting continuous severity score. *Metab Clin Exp.* (2014) 63:218–25. 10.1016/j.metabol.2013.10.006 24290837 PMC4071942

[25] Gurka MJ Ice CL Sun SS Deboer MD. A confirmatory factor analysis of the metabolic syndrome in adolescents: an examination of sex and racial/ethnic differences. *Cardiovasc Diabetol.* (2012) 11:128. 10.1186/1475-2840-11-128 23062212 PMC3489601

[26] Ford ES Li C Cook S Choi HK. Serum concentrations of uric acid and the metabolic syndrome among US children and adolescents. *Circulation.* (2007) 115:2526–32. 10.1161/CIRCULATIONAHA.106.657627 17470699

[27] Zimmet P Alberti G Kaufman F Tajima N Silink M Arslanian S The metabolic syndrome in children and adolescents. *Lancet.* (2007) 369:2059–61. 17586288 10.1016/S0140-6736(07)60958-1

[28] DeBoer MD Gurka MJ. Ability among adolescents for the metabolic syndrome to predict elevations in factors associated with type 2 diabetes and cardiovascular disease: data from the national health and nutrition examination survey 1999-2006. *Metab Syndr Relat Disord.* (2010) 8:343–53. 10.1089/met.2010.0008 20698802 PMC3046372

[29] Lee S Bacha F Gungor N Arslanian S. Comparison of different definitions of pediatric metabolic syndrome: relation to abdominal adiposity, insulin resistance, adiponectin, and inflammatory biomarkers. *J Pediatr.* (2008) 152:177–84. 10.1016/j.jpeds.2007.07.053 18206686

[30] Reinehr T de Sousa G Toschke AM Andler W. Comparison of metabolic syndrome prevalence using eight different definitions: a critical approach. *Arch Dis Child.* (2007) 92:1067–72. 10.1136/adc.2006.104588 17301109 PMC2066078

[31] Jin J Dalwadi SM Masand RP Hall TR Anderson ML Ludwig MS. Association between metabolic syndrome and endometrial cancer survival in a SEER-medicare linked database. *Am J Clin Oncol.* (2020) 43:411–7. 10.1097/coc.0000000000000686 32205571

[32] Ni J Zhu T Zhao L Che F Chen Y Shou H Metabolic syndrome is an independent prognostic factor for endometrial adenocarcinoma. *Clin Transl Oncol.* (2015) 17:835–9. 10.1007/s12094-015-1309-8 26260911

[33] Alberti KG Eckel RH Grundy SM Zimmet PZ Cleeman JI Donato KA Harmonizing the metabolic syndrome: a joint interim statement of the international diabetes federation task force on epidemiology and prevention; national heart, lung, and blood institute; American heart association; world heart federation; international atherosclerosis society; and international association for the study of obesity. *Circulation.* (2009) 120:1640–5. 10.1161/circulationaha.109.192644 19805654

[34] Zheng R Li M Xu M Lu J Wang T Dai M Chinese adults are more susceptible to effects of overall obesity and fat distribution on cardiometabolic risk factors. *J Clin Endocrinol Metab.* (2021) 106:e2775–88. 10.1210/clinem/dgab049 33570562

[35] Lu J Wang L Li M Xu Y Jiang Y Wang W Metabolic syndrome among adults in China: the 2010 China noncommunicable disease surveillance. *J Clin Endocrinol Metab.* (2017) 102:507–15. 10.1210/jc.2016-2477 27898293

[36] Vitale SG Capriglione S Zito G Lopez S Gulino FA Di Guardo F Management of endometrial, ovarian and cervical cancer in the elderly: current approach to a challenging condition. *Arch Gynecol Obstet.* (2019) 299:299–315. 10.1007/s00404-018-5006-z 30542793

[37] Siesto G Uccella S Ghezzi F Cromi A Zefiro F Serati M Surgical and survival outcomes in older women with endometrial cancer treated by laparoscopy. *Menopause.* (2010) 17:539–44. 10.1097/gme.0b013e3181c4e9f5 20032796

[38] Vitale SG Valenti G Gulino FA Cignini P Biondi A. Surgical treatment of high stage endometrial cancer: current perspectives. *Updates Surg.* (2016) 68:149–54. 10.1007/s13304-015-0340-1 26826083

[39] Olson SH De Vivo I Setiawan VW Lu KH. Symposium on advances in endometrial cancer epidemiology and biology. *Gynecol Oncol.* (2015) 138:497–500. 10.1016/j.ygyno.2015.07.106 26232339 PMC4910514

