# Supplementary figures and images for: Prognostic-Related Metabolic Score for Survival Prediction in Early-Stage Endometrioid Endometrial Cancer: A Multi-Center and Retrospective Study
Source: Front Med (Lausanne). 2022 Apr 28;9:830673. doi: 10.3389/fmed.2022.830673 (PMC9096267; doi:10.3389/fmed.2022.830673)

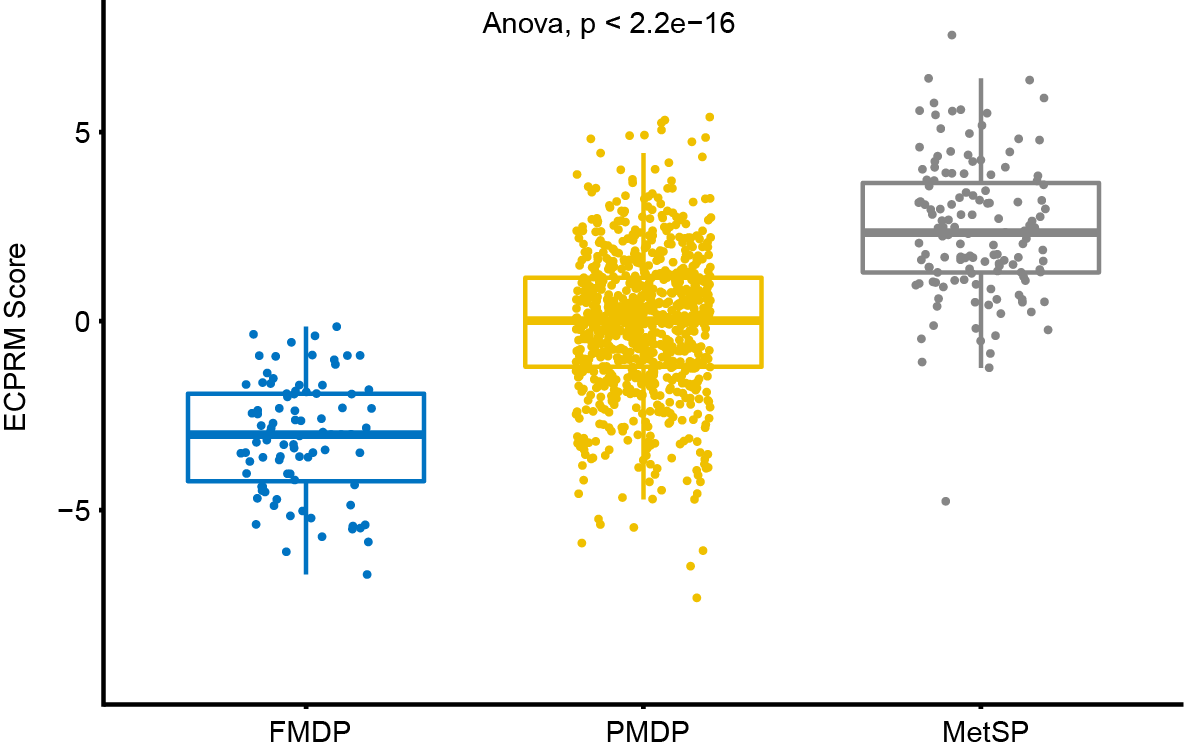

Supplement: Supplementary Figure 1 — ANOVA analysis revealed significant differences in endometrial cancer prognostic-related metabolic (ECPRM) Score of the free metabolic disorder in patient (FMDP), partial metabolic disorder patient (PMDP), and metabolic syndrome in patient (MetSP) group in the trainset. [file Image_1.TIF]
