# Supplementary material for: Prognostic-Related Metabolic Score for Survival Prediction in Early-Stage Endometrioid Endometrial Cancer: A Multi-Center and Retrospective Study
Source: Front Med (Lausanne). 2022 Apr 28;9:830673. doi: 10.3389/fmed.2022.830673 (PMC9096267; doi:10.3389/fmed.2022.830673)
Supplement: Supplementary file 2 [file Table_1.docx]

**Table S1 Confirmatory Factor Analysis Results**

|  | | Trainset (n=998) |
| --- | --- | --- |
| Akaike’s Information Criteria (AIC) | | 14028.864 |
| Root Mean Square Error of Approximation (RMSEA) | | 0.042 |
| Standardized Root Mean Square Residual (SRMR) | | 0.017 |
| Comparative of Fit Index (CFI) | | 0.976 |
| Factor Loadings | |  |
| BMI | | 0.999 |
| SBP | | 0.999 |
| HDL | | 0.780 |
| Triglycerides | | 0.835 |
| BG | | 0.999 |
| ECPRM Score | 0.2500805* BMI +0.05745441* SBP +0.2553792* BG +  1.164205* log2(triglycerides) -1.844085* HDL -14.16841 | |

.
